# Detecting stereoscopic distribution of carbon dioxide using single photon spectroscopy

**DOI:** 10.1016/j.fmre.2025.01.016

**Published:** 2025-02-13

**Authors:** Youwen Sun, Xiaomin Hu, Pan Yu, Biheng Liu, Jian Li, Yunsheng Dong, Ke Wang, Yanrui Xu, Haobin Zhao, Cheng Liu

**Affiliations:** a Key Laboratory of Environmental Optics and Technology, Anhui Institute of Optics and Fine Mechanics, HFIPS, Chinese Academy of Sciences, Hefei 230031, China; b CAS Key Laboratory of Quantum information, University of Science and Technology of China, Hefei 230026, China; c College of Control Science and Engineering, Zhejiang University, Hangzhou 310027, China; d Institute of quantum information and technology, Nanjing University of Posts and Telecommunications, Nanjing 210003, China; e Department of Precision Machinery and Precision Instrumentation, University of Science and Technology of China, Hefei 230026, China; f Key Laboratory of Precision Scientific Instrumentation of Anhui Higher Education Institutes, University of Science and Technology of China, Hefei 230026, China

**Keywords:** Single photon, LIDAR, Atmospheric detection, Greenhouse gases, Climate change

## Abstract

It is of great significance to develop a high-precision and high spatiotemporal resolution of stereoscopic detection technology to finely delineate the horizontal and vertical distributions of CO_2_. This study elaborates on the superiorities of single photon spectroscopy, key steps to develop a high-performance single photon LIDAR, and their potential applications. Single-photon CO_2_ LIDAR integrates quantum sensing techniques with classical spectral measurement techniques, and can overcome the detection limitations of traditional CO_2_ LIDAR in terms of detection distance, sensitivity, and spatiotemporal resolution. We anticipate that the single-photon spectroscopic technology can be widely applied in the detection of regional distribution and emission fluxes of CO_2_, transboundary transport fluxes of CO_2_, and in supporting the establishment of a new carbon monitoring framework.

## 1 Introduction

Carbon monitoring technology plays a key role in assessing carbon emissions, and formulating and adjusting emission reduction strategies. Carbon emission sources are complex, diverse, and variable. Carbon dioxide (CO_2_) level at a specific place is determined by both local emissions and long-range transport from external sources. Long-range transport not only occurs near the ground but also at high altitudes. It is of great significance to develop a high-precision and high spatiotemporal resolution of stereoscopic detection technology to finely delineate the horizontal and vertical distributions of CO_2_. On the one hand, high-precision and high spatiotemporal resolution of stereoscopic detection technology enables accurately locating carbon emission sources, quantifying emission scales, and clarifying transport pathways and relative contributions, which results in more effective formulation and implementation of emission reduction measures. On the other hand, by accurately measuring the stereoscopic distribution of CO_2_, it is possible to simulate and predict climate change trends more accurately, support in-depth research in atmospheric science, meteorology, and environmental science, and enhance the understanding of atmospheric processes.

At present, there is a variety of active and passive detection technologies deployed on various platforms to monitor CO_2_ globally, which have played an important role in carbon attribution and accounting. However, all these detection technologies mainly rely on the traditional optoelectronic system, and both the light source and the signal detection means are based on the collective effect of photons, and cannot reach the level of individual photons. In such cases, the detection performance under extremely weak light signals and high noise is poor, and the detection distance, accuracy, and spatiotemporal resolution cannot be beyond the limit of classical detection technology.

Based on quantum mechanics established in the early 20th century, scientists have begun to reveal the quantum properties of matter and apply this knowledge to technological and industrial innovation. Technologies such as semiconductor physics, laser technology, and nuclear magnetic resonance have been developed by relying on the collective effect of the ensemble system operation, which has greatly promoted the progress of science and industry. Compared with the traditional optoelectronic detection technology produced by the first quantum revolution, the quantum information technology produced by the second quantum revolution can manipulate and measure individual quantum (such as individual photons or individual particles), making the detection accuracy of the basic physical quantity reach the quantum limit [1]. Single quantum manipulation and detection technology can capture extremely low light intensity, i.e., signals at the level of individual photons - the smallest unit of energy distribution. Compared with classical optoelectronic technology, single quantum technology has advantages such as high sensitivity, high time resolution, and high signal-to-noise ratio [[2], [3], [4]]. Given its unique manipulation and measurement methods, it will bring new functions and performance that classical technology cannot compare to new types of computing and sensing methods. For example, single quantum manipulation and detection technology has been widely applied in the field of quantum imaging, surpassing the imaging resolution and contrast limit of traditional optoelectronic technology, and showing a strong application prospect.

Photons are excellent carriers for detecting atmospheric spectral structure, and the distributions of atmospheric compositions can be finely resolved by analyzing the interaction between photons and the atmosphere. However, single quantum manipulation and measurement technology is still in its infancy in the field of atmospheric spectral detection. The integration of quantum precision measurement technology with classical environmental spectral measurement technology is expected to break through the bottleneck of classical optical measurement technology, resulting in a new disruptive carbon monitoring technology with high sensitivity and high vertical resolution.

This study elaborates on the superiorities of single photon spectroscopy with respect to the classical spectral technology, and key steps to develop a high-performance single photon LIDAR. Potential applications of single-photon LIDAR and challenges in practical application are also discussed.

## 2 Principles and advantages of single photon spectroscopy

The single-photon CO_2_ LIDAR operates on the principle of Differential Absorption LIDAR (DIAL), which involves emitting two pulsed waves with slightly different wavelengths, one of which is absorbed by CO_2_ while the other is not. The on-line wavelength is near the center of the CO_2_ absorption line and is primarily attenuated by CO_2_ absorption, while the off-line wavelength is far from the center and only weakly attenuated. The difference in the backscattered signals from these wavelengths provides range-resolved measurements of CO_2_ concentration. For the detection of atmospheric constituents using LIDAR technology, the signal-to-noise ratio (SNR) of the detected signal can be expressed as:

(1) $$SNR\left(R\right)\propto E\times A\times \frac{\eta_{q}}{hv}\times \frac{1}{N_{d}}\times \frac{1}{R^{2}}$$

where *R, E*, A, *η_q_*, h*, v*, and *N_d_* represent the distance, the energy of the emitted laser, the area of the telescope, the quantum conversion efficiency, the Planck constant, the frequency of the received laser and the noise of the detector, respectively.

The traditional LIDAR technology relies on the collective effect of optoelectronic detection. It usually improves the SNR of the signal by increasing the energy of the emitted laser E and the aperture of the telescope A. To detect the vertical distribution of CO_2_ over a range of several kilometers, laser energy of 5–10 mJ and a telescope with a diameter of several meters are required [2,4]. Representative research teams that detect CO_2_ variability using traditional LIDAR technology are from institutions such as the German Aerospace Center (DLR), the French National Space Agency, the National Institute of Standards and Technology (NIST) in the United States, the University of Science and Technology of China, the Anhui Institute of Optics and Fine Mechanics of the Chinese Academy of Sciences, and Wuhan University. High laser energy and large-aperture telescopes lead to very large detection systems and high production costs. In contrast, the single-photon LIDAR technology improves the SNR by increasing quantum efficiency *η_q_*, reducing detection noise *N_d_*, and detection energy *hv*. Integrating quantum sensing techniques with single-photon CO_2_ LIDAR enhances its capabilities by leveraging high-sensitivity single-photon detectors (SNSPDs), which can detect individual photons and offer high timing accuracy, high multicount rate, and a wide response band. The integration of SNSPDs with LIDAR systems allows for the detection of even the faintest signals, improving the detection range, depth accuracy, and acquisition time of single-photon LIDAR. Furthermore, the use of quantum light sources and quantum interferometry can effectively improve the sensitivity and signal-to-noise ratio of CO_2_ detection, such as using quantum entanglement as the light source and utilizing two-photon interference effects to achieve high-precision detection of CO_2_ and wind speed. For example, using quantum upconversion technology and two-photon Hong-Ou-Mandel (HOM) interferometry technology with the same telescope aperture, we only need an order of magnitude lower output power to achieve the same wind measurement effect [5]. This method can also be effectively transplanted into the application of CO_2_ radar, and it is expected to effectively increase the sensitivity and detection range of CO_2_ radar. Relevant experiments have been verified in desktop experiments [6].

Compared to traditional LIDAR technology, the single-photon LIDAR technology has the following significant advantages: (1) Sensitivity down to quantum-limit: Single-photon detection technology can detect signal strengths at an individual photon level [7], capturing extremely weak photon echoes and enhancing the ability to detect CO_2_ with lower variability. (2) High spatiotemporal resolution: Single-photon LIDAR technology accurately measures the arrival time of each photon, providing high-precision temporal photon data [8]. High spatial resolution allows for resolving CO_2_ concentration distribution in both vertical and horizontal directions, which is crucial for understanding the emission, diffusion, and exchange processes of CO_2_. (3) Enhanced penetration capability: The high sensitivity and strong penetration ability of single-photon LIDAR technology enable it to work effectively under various environmental and weather conditions, including low visibility conditions under clouds and fog [9]. (4) Longer detection range and greater flexibility: Single-photon LIDAR technology can capture much weaker photon echoes and have a more transportable design than traditional LIDAR technology [10]. As a result, with the same emitted laser power and telescope aperture, a single-photon LIDAR is capable of detecting CO_2_ over a longer distance. (5) Real-time data processing and analysis: Highly accurate time recording and data processing capabilities enable single-photon LIDAR technology to provide near real-time data analysis, which is valuable for quickly responding to environmental changes and making policy decisions.

## 3 Steps to develop a high-performance single photon LIDAR

**Fig. 1:**
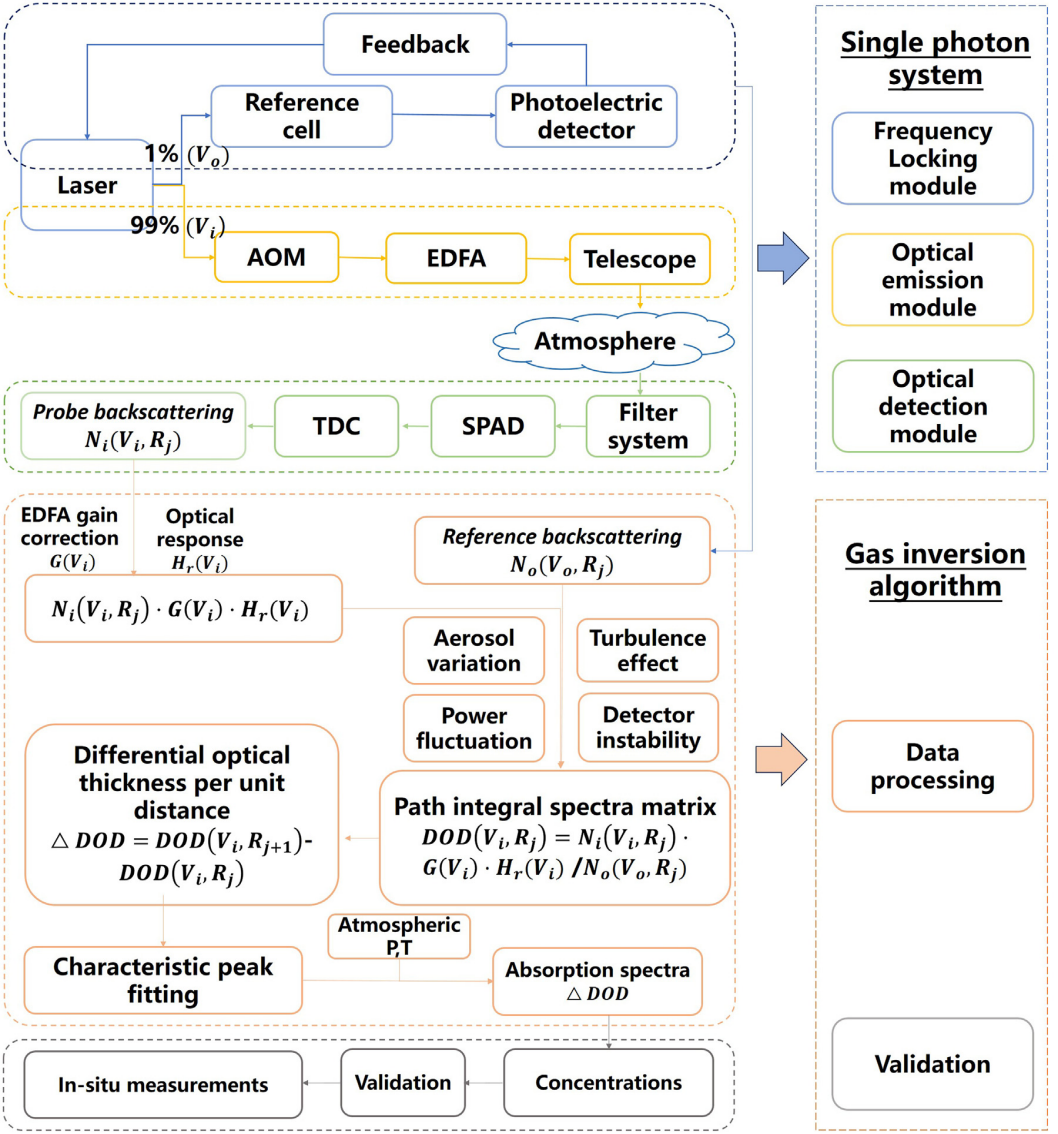
**Diagram of a single-photon LIDAR and its retrieval algorithm.** AOM, EDFA, SPAD, and TDC represent acousto-optic modulator, erbium-doped fiber amplifier, single-photon avalanche diode, and time-to-digital converter, respectively.

Developing a single-photon LIDAR for stereoscopic detection of CO_2_ typically involves four steps: First, experimental simulations are conducted to provide theoretical guidance for the development of single-photon detection equipment and the inversion algorithms. Second, key technologies such as light source emission, frequency locking, and single-photon reception and processing are addressed to develop the equipment for single-photon detection equipment. Third, key technologies such as background subtraction, cross-interference correction, and characteristic peak fitting are resolved to develop the single-photon inversion algorithm. Finally, application demonstrations are carried out in key areas, where high-precision and high-spatiotemporal resolution of stereoscopic CO_2_ distribution are obtained through sensitivity testing and in-situ calibration. Diagram of a typical single-photon LIDAR and its retrieval algorithm is shown in Fig. 1. We elaborate them as follows:

### 3.1 Experimental simulation for single photon detection

The experimental simulation research that should be conducted before equipment construction includes (a) analysis of the impact of gas interference, optical thickness, temperature sensitivity, and the advantages and disadvantages of characteristic absorption spectra to optimize the designated detection waveband; (b) simulation of single-photon LIDAR echo signals and noise reduction to explore the feasibility of single-photon stereoscopic detection of CO_2_ in the atmosphere; (c) simulation of hybrid spectra and non-uniform concentration distribution, comparison of spectral inversion using area method and lookup table method, and using different fitting models.

### 3.2 Development of single-photon detection equipment

Single-photon detection equipment can be constructed with theoretical guidance from the aforementioned simulation research. The key components to be constructed include (a) development of the light source emission system, mainly addressing technical challenges such as noise suppression, time-division multiplexing of detection light and reference light, and real-time calibration; (b) development of the frequency locking system, mainly addressing technical challenges such as frequency comb repetition and frequency locking, lock frequency feedback design, and adaptive frequency scanning and locking; (c) development of the single-photon reception and data processing system, mainly for the design of the single-photon detection system, data reception system, and data processing procedures.

### 3.3 Development of a single-photon retrieval algorithm

Guided by the conclusion of simulation research, the single-photon retrieval algorithm for stereoscopic CO_2_ distribution is developed, including (a) frequency correction, background subtraction of single-photon echo signals, and corrections for atmospheric turbulence-induced aerosol changes, laser power fluctuations, detector instability, and instability of signal coupling efficiency; (b) algorithms for cross-interference correction, temperature and pressure correction of spectral line shapes, and single-photon frequency sweeping iterative fitting of characteristic peaks to calculate the spatiotemporal distribution of CO_2_.

### 3.4 Demonstration and verification

Single-photon carbon three-dimensional distribution detection technology verification and application demonstrations can be carried out in key cities or industrial parks, including (a) assessing the impact of frequency scanning intervals, frequency locking and non-locking, quantum fluctuations, and different atmospheric environments on the results of single-photon carbon three-dimensional distribution detection; (b) placing high-precision in-situ greenhouse gas analyzers at different distances to verify the results of single-photon measurements with in-situ measurement results and perform error analysis; (c) studying regional carbon distribution and emission fluxes based on the results of single-photon detection.

## 4 Potential applications of single-photon LIDAR

Given the superiorities in detection range, sensitivity, and spatiotemporal resolution, single-photon LIDAR for stereoscopic carbon detection is expected to become a powerful tool for investigating and monitoring the stereoscopic distribution and variability of regional CO_2_, with the following three major application prospects anticipated.

### 4.1 Detection of regional distribution and emission fluxes of CO_2_

**Fig. 2:**
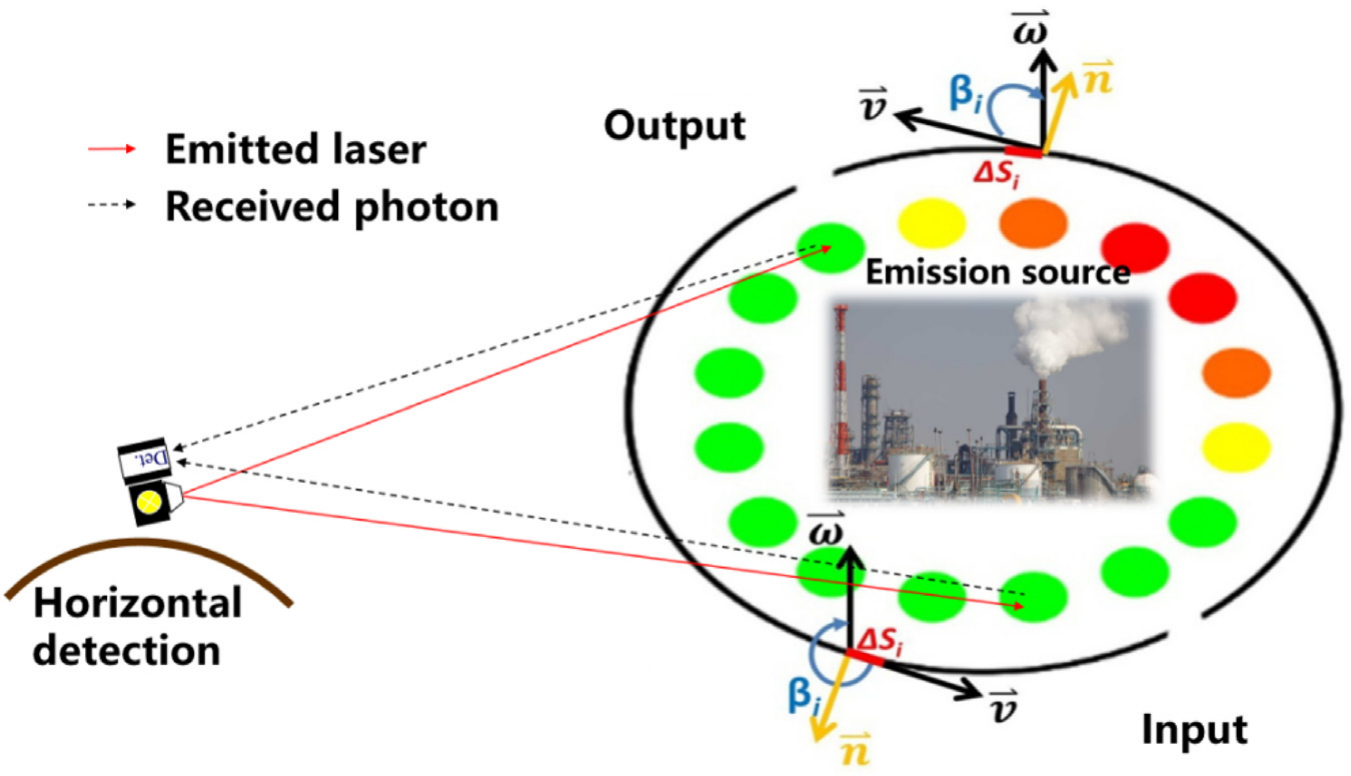
**Single-photon LIDAR concept for detecting regional distribution and emission flux of CO_2_**.

As shown in Fig. 2, deploying a single-photon LIDAR at the top of mountains or buildings, the spatial distribution of regional CO_2_ can be derived by scanning the emission source with a full-range horizontal sweep (Fig. 1). By combining the measurements with the horizontal meteorological field, it is possible to calculate the emission fluxes of the CO_2_ source. This application aspect provides novel technical support for accurately implementing the “pollution reduction and carbon reduction” strategy [11]. A recent example that uses single-photon LIDAR to derive regional distribution of CO_2_ was demonstrated in Yu et al. (2021), which detected CO_2_ in the ambient atmosphere with uncertainty as low as ± 1.2% at a spatial resolution of 60 m and a time resolution of 10 min. Compared to the classical technologies that obtain only column-integrated concentration, the spatial resolution is improved by 2–3 orders of magnitude. Furthermore, a detect range of 6 km has been achieved with laser energy in the μJ range and the diameter of a telescope in the cm range [8]. However, the maximum detection range of a classical LIDAR is only several hundred meters with similar laser energy and telescope aperture.

### 4.2 Detection of transboundary transport fluxes of CO_2_

**Fig. 3:**
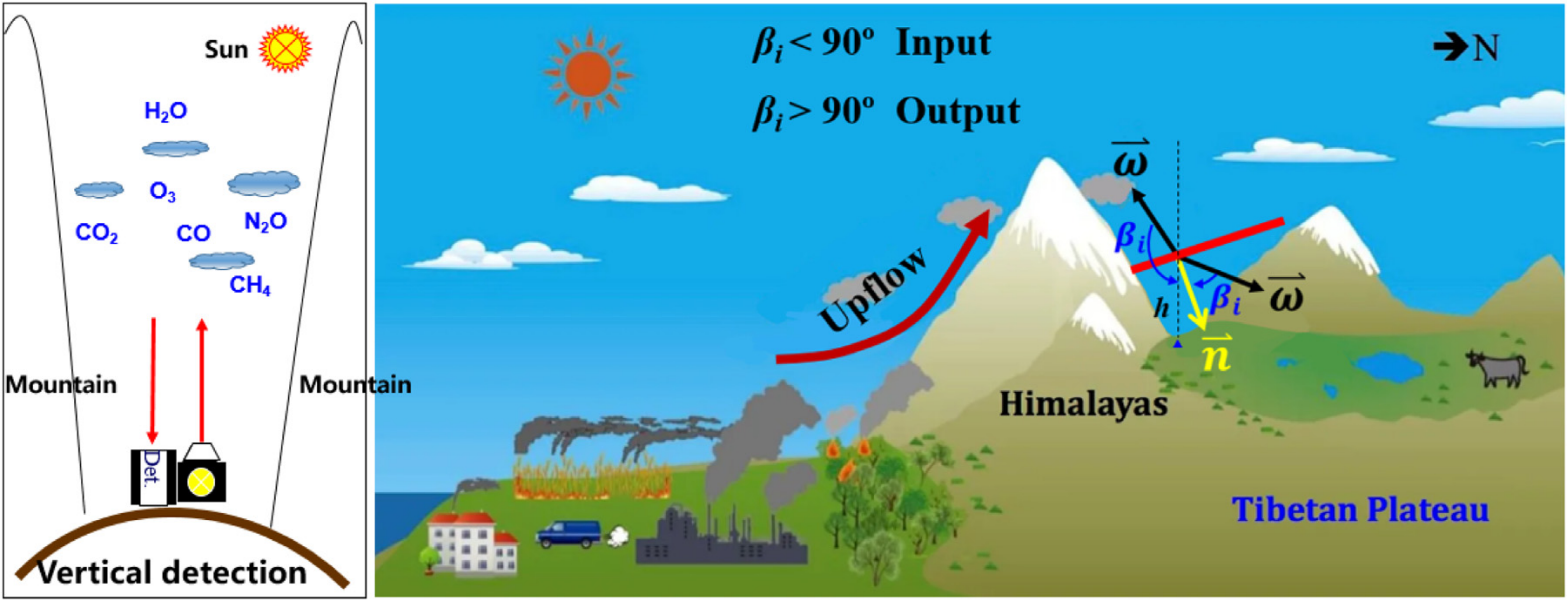
**Single-photon LIDAR concept for detecting transboundary transport flux of CO_2_**.

By detecting the vertical distribution of CO_2_ in the transport pathways and coupling it with vertical meteorological data, the transboundary flux of CO_2_ can be inferred [12]. For example, as shown in Fig. 3, driven by the Asian monsoon, greenhouse gases from South Asia can be transported into the Tibetan Plateau along the Himalayan Valley. In this case, a single-photon LIDAR deployed at the bottom of the Himalayan valley is capable of deriving the carbon flux transported from South Asia to the Tibetan Plateau along a specific pathway. Similarly, single-photon LIDAR can also measure the cross-border transport flux between different industrial regions.

### 4.3 A new carbon monitoring framework

**Fig. 4:**
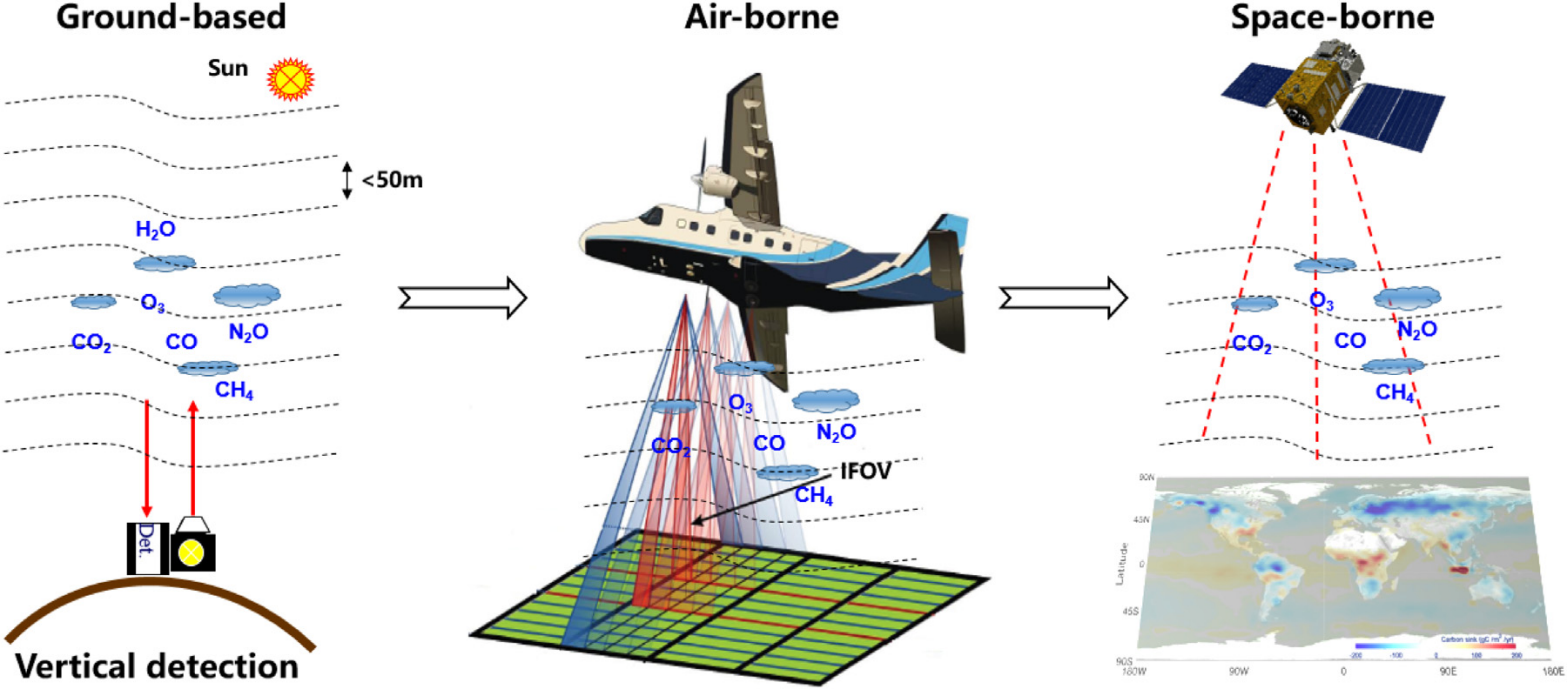
**Single-photon LIDAR concept for building a new carbon monitoring framework**.

As shown in Fig. 4, by conducting ground-based exploration and airborne validation, a foundation can be laid for the development of highly sensitive and high-spatiotemporal resolution of space-borne single-photon carbon LIDAR. Establishing a new “ground-air-space” single-photon carbon monitoring technological framework [13] can promote a leapfrog development in global carbon accounting technology and carbon source/sink research. Deploying a constellation of single-quantum carbon monitoring satellites enables the real-time acquisition of global carbon distribution with high sensitivity and high vertical resolution, which is of great significance for clarifying global carbon source/sink information, understanding the patterns of evolution, resolving disputes over global carbon accounting, and securing the discourse power in carbon accounting.

## 5 Potential challenges in practical application

In practical application, while the single-photon LIDAR technology tends to have numerous advantages, it is prudent to consider potential uncertainties and limitations that may arise. Only by overcoming all challenges through rigorous testing, technological refinement, and strategic planning for deployment, can the technology realize its full potential and contribute effectively to the monitoring and management of carbon emissions. We elaborate on some challenges as follows.

Firstly, the technology’s sensitivity to environmental conditions cannot be overlooked. Single-photon LIDAR operates optimally under controlled conditions, but in the field, it may face challenges such as extreme weather, which could affect the stability and accuracy of the system. High winds, heavy rain, and fog could potentially degrade the signal inconsistency and penetration capability of the LIDAR, thus impacting the quality of data collected.

Secondly, the maturity of the technology at its current stage is a significant factor. Single-photon LIDAR is an emerging technology, and while it shows great promise, it may not yet be fully ready for widespread deployment. The technology’s reliability over long periods and in various operational conditions requires further validation through extensive field testing and experimentation.

Thirdly, the integration of quantum sensing techniques with classical spectral measurement techniques presents its own set of challenges. The complexity of the system could lead to increased susceptibility to errors, particularly in the precision of frequency locking and the accuracy of single-photon detection. The new principles of quantum laser radar, such as HOM interference or squeezed states of light technology, often require more complex technical means compared to traditional radar, which brings difficulties in radar stability and cost. Ensuring the robustness of these components under real-world conditions is crucial for the technology’s success.

Fourthly, the scalability and cost-effectiveness of single-photon LIDAR systems are also considerations. While the technology promises high sensitivity and resolution, the production and maintenance costs of such systems could be prohibitive, especially for large-scale deployment. Balancing the benefits of high-performance detection with economic feasibility is a critical challenge.

## 6 Conclusion and outlook

Traditional CO_2_ LIDAR improves the SNR of the detected signal by increasing laser energy and telescope aperture, and the detection limits such as distance, sensitivity, and spatiotemporal resolution are constrained by the technological bottleneck of photon collective effects. Single-photon CO_2_ LIDAR integrates quantum precision measurement techniques with classical spectral measurement techniques, and by addressing key technologies such as the detection of weak single-photon signals, background interference subtraction, and single-photon retrieval, it can overcome the detection limitations of traditional CO_2_ LIDAR in terms of detection distance, sensitivity, and spatiotemporal resolution. We anticipate that the single-photon spectroscopic technology can be widely applied in the detection of regional distribution and emission fluxes of CO_2_, transboundary transport fluxes of CO_2_, and in supporting the establishment of a new carbon monitoring framework.

Although single-photon LIDAR has already achieved a certain degree of quantum enhancement, its potential in single-quantum measurements is far from being fully exploited. In the future, to fully utilize the advantages of quantum sensing, it will be necessary not only to advance detection technology to the single-quantum level but also to employ quantum light sources [14]. For instance, entangled light sources are a core type of quantum light source that allows photons separated by great distances to remain connected, enabling the behavior of one photon to instantly affect another photon with which it is entangled [15]. Unlike traditional light sources, entangled light sources exhibit non-classical effects such as quantum non-locality and strong correlations. These effects can surpass the limitations of classical spectroscopic techniques. For example, using non-locality, it is possible to conduct non-contact spectral measurements, where spectral information can be obtained without the need for photons to interact with the target gas, simply by detecting the entangled photons [16]. Additionally, the strong correlations allow for differential measurements between two photons, achieving spectral detection below the shot noise limit [17]. The quantum spectral sensing technology that integrates quantum light source and quantum detection is expected to break through the technical bottleneck of classical optical measurement, significantly improve detection sensitivity, accuracy, spatiotemporal resolution and other performance, achieve spectral detection close to the quantum limit, and promote the leapfrog development of environmental measurement technology.

## CRediT authorship contribution statement

YS and XH designed the study and wrote the paper. The other authors provided constructive comments.

## Declaration of competing interest

The authors declare that they have no conflicts of interest in this work.
